# Disparities by Sex and Race and Ethnicity in Death Rates Due to Opioid Overdose Among Adults 55 Years or Older, 1999 to 2019

**DOI:** 10.1001/jamanetworkopen.2021.42982

**Published:** 2022-01-11

**Authors:** Maryann Mason, Rebekah Soliman, Howard S. Kim, Lori Ann Post

**Affiliations:** 1 Department of Emergency Medicine, Northwestern University Feinberg School of Medicine, Chicago, Illinois; 2 Buehler Center for Health Policy and Economics, Northwestern University, Chicago, Illinois; 3 currently an undergraduate student at Northwestern University, Weinberg College of Arts and Sciences, Evanston, Illinois; 4 Center for Health Services and Outcomes Research, Northwestern University Feinberg School of Medicine, Chicago, Illinois; 5 Associate Editor, *JAMA Network Open*

## Abstract

**Importance:**

Rates of opioid overdose deaths are increasing for older adults. Less is known about these deaths compared with those of younger adults.

**Objective:**

To analyze rate variation among opioid overdose deaths in older adults by sex and by race and ethnicity over time.

**Design, Setting, and Participants:**

This 21-year longitudinal cross-sectional study of adults who died due to opioid overdose at 55 years or older stratified by sex and by race and ethnicity used data from the Multiple Cause of Death database from the Centers for Disease Control and Prevention Wide-Ranging Online Data for Epidemiologic Research. The data include all opioid overdose deaths among this age group that occurred between January 1, 1999, and December 31, 2019 (N = 79 893).

**Exposures:**

Sex and racial and ethnic groups.

**Main Outcomes and Measures:**

Rates of opioid overdose deaths per 100 000 population by sex and by race and ethnicity for persons 55 years or older.

**Results:**

During the period 1999 to 2019, 79 893 US residents 55 years or older died due to an opioid overdose. Among these individuals, 79.97% were aged 55 to 64 years, and 58.98% were men. Annual numbers of deaths increased over time from 518 in 1999 to 10 292 in 2019. Annual rates of opioid overdose deaths per 100 000 persons 55 years or older increased over time and ranged from 0.90 in 1999 to 10.70 in 2019. Substantial variation by sex and by race and ethnicity was found. In 2013, rates among non-Hispanic Black men began to diverge from those of other demographic subgroups. By 2019, the opioid overdose fatality rate among non-Hispanic Black men 55 years or older was 40.03 per 100 000 population, 4 times greater than the overall opioid overdose fatality rate of 10.70 per 100 000 for persons of the same age.

**Conclusions and Relevance:**

In this longitudinal cross-sectional study of US adults who died due to opioid overdose, the burden of opioid overdose deaths among older adults since 2013 was most concentrated among non-Hispanic Black men. Deaths among non-Hispanic Black men were disproportionality represented in the overall increase in the rate of opioid overdose deaths among older adults. Further research is needed to inform policy and practice.

## Key Points

**Question:**

Is there variation by sex and by racial and ethnic subgroups in rates of opioid overdose deaths among older adults over time?

**Findings:**

In this cross-sectional study of opioid overdose deaths in older adults from 1999 to 2019, 79 893 persons in the US aged 55 years or older died due to opioid overdose. The annual overall death rate per 100 000 persons for this population ranged from a low of 0.9 in 1999 to a high of 10.7 in 2019 and increased annually from 2000 on; rates varied substantially by sex and by race and ethnicity over time.

**Meaning:**

These results suggest a need for increased screening for substance use disorder among older adults along with outreach and treatment models adapted to their unique circumstances.

## Teaser

This cross-sectional study identifies potential divergences by race and ethnicity and by sex among older US adults who died due to opioid overdose.

## Introduction

The US has been gripped by an opioid crisis for at least the last 2 decades.^1,2^ Over time, the crisis has evolved in terms of the population segments most severely affected. In recent years, there has been a notable increase in rates of opioid overdose deaths for adults 55 years or older.^3^ The number and proportion of adults 65 years and older with substance use disorder (SUD) is increasing.^4^ The average age of admissions for substance use treatment has also increased.^5^ All these factors coincide with the aging of the American population.^6^

The evidence base regarding opioid use disorder (OUD) among older adults is limited.^7^ Emergency department visits for opioid misuse increased 220% from 2006 to 2014 for persons 65 years and older, an indicator of a growing problem.^8^ Factors associated with OUD among older adults include an increased number of chronic conditions, polypharmacy, and greater injury risk due to falls and fractures.^8^ We know less about factors specifically associated with opioid overdose deaths among older adults.^9^ The prevalence of chronic conditions treated with opioids such as arthritis and cancer increases with age, so exposure to medically prescribed opioids among older adults is higher, thus the potential risk for fatal overdose in this population increases.^10,11,12^ Aging decreases the body’s ability to metabolize opioids, a potential contributor to fatal overdoses.^13,14^ A decline in cognitive functioning may interfere with taking opioids as prescribed, again a potential contributor to fatal overdoses.^15^ Social isolation and depression increase with age and are associated with SUD, which may play a role in opioid overdose deaths among older adults.^16,17^ In addition, the present generation of older adults uses substances at higher rates than previous generations.^4,18^ The higher prevalence may contribute to opioid overdose deaths in older adults in combination with physical changes associated with aging.^4^

In general, during the last 20 years, men have consistently had higher rates of opioid overdose deaths compared with women.^19^ Racial and ethnic group disparities in rates of opioid drug overdose deaths have fluctuated between 1999 and 2019. As the epidemic escalated, rates for non-Hispanic White adults rose exponentially compared with other groups, driving up the overall rate of opioid overdose deaths. However, from 2017 to 2019 (the latest year for which data are available), the gap between non-Hispanic Black and non-Hispanic White adults has narrowed as rates rose for the former group and modestly declined for the latter group.^20^ We hypothesize that the patterns of the overall rate of opioid overdose fatalities by sex and by race and ethnicity would be consistent with those for opioid overdose fatalities among older adults.

To advance research into recent increases in opioid overdose deaths in older adults, we compared fatality rates between 1999 and 2019 by sex and by race and ethnicity. Our objective was to identify potential divergence by race and ethnicity and by sex characteristics compared with the overall pattern of increasing rates of opioid overdose deaths among older adults over time.

## Methods

Data for this cross-sectional study were downloaded into Excel, version 2016 (Microsoft Corporation) from the Centers for Disease Control and Prevention Wide-Ranging Online Data for Epidemiologic Research (WONDER) database.^21^ The study used publicly available deidentified data and was determined exempt from approval and informed consent by the Northwestern University Institutional Review Board. This study followed the Strengthening the Reporting of Observational Studies in Epidemiology (STROBE) reporting guideline.

Case inclusion criteria included death between January 1, 1999, and December 31, 2019, with an underlying cause of death identified by *International Statistical Classification of Diseases and Related Health Problems, Tenth Revision*, codes X40 to X44 (unintentional), X60 to X64 (suicide), X85 (homicide), or Y10 to Y14 (undetermined) and a multiple cause of death code of T40.1 (opium), T40.2 (heroin), T40.3 (other opioids), T40.4 (other synthetic narcotics), or T40.6 (other and unspecified narcotics).^22^

US rates were downloaded from the WONDER database for adults 55 years or older, by sex (male or female), race (American Indian or Alaska Native, Asian or Pacific Islander, Black, or White), and ethnicity (Hispanic or Latino or non-Hispanic or non-Latino).^21^ Race and ethnicity in the WONDER database are ascertained from death certificates. We included sex and race and ethnicity to identify potential disparities in rates of opioid overdose fatalities. We examined rates for the following 10 subgroups: Hispanic or Latina women; Hispanic or Latino men; non-Hispanic American Indian or Alaska Native, non-Hispanic Asian or Pacific Islander, non-Hispanic Black, and non-Hispanic White men; and non-Hispanic American Indian or Alaska Native, non-Hispanic Asian or Pacific Islander, non-Hispanic Black, and non-Hispanic White women. We generated line graphs depicting annual rates of opioid overdose deaths for those 55 years and older from 1999 to 2019 and then by sex and by race and ethnicity. We compared the range of annual rates of opioid overdose deaths among sex and by race and ethnicity and examined change over time within and between subgroups. We used adjusted rates of overdose deaths for each subgroup based on National Center for Health Statistics population estimates for non-Census years and population figures from the US Census Bureau for 2000 and 2010 available through WONDER.^21^ Because we report data on all opioid overdose deaths among the US population, we did not calculate 95% CIs for any population estimates. To estimate similarity of trajectories, we ran a multivariate regression model with year as the independent variable and the 10 subgroups as dependent variables to produce parameter estimates, including slope (B). We used Excel, version 2016 (Microsoft Corporation), and SPSS, version 27 (IBM Corporation), for analysis and graph production. Two-sided *P* < .05 indicated significance.

## Results

**Figure 1.**
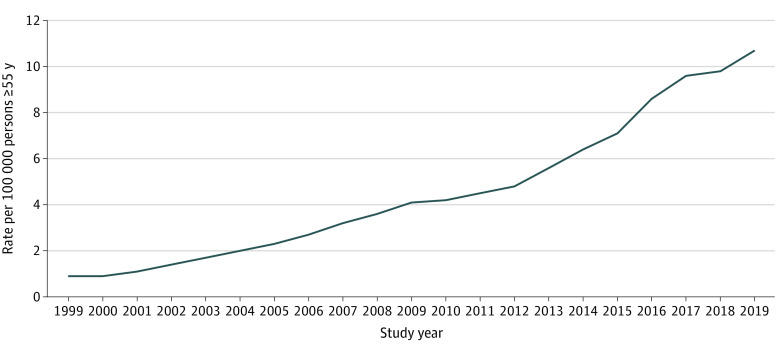
Rates of Opioid Overdose Deaths per 100 000 Persons 55 Years and Older, 1999 to 2019

During the period 1999 to 2019, 79 893 US residents 55 years or older died due to an opioid overdose. Most of these individuals (79.97%) were aged 55 to 64 years; 58.98% were men and 41.01% were women. The annual number of deaths ranged from a low of 518 in 1999 to a high of 10 292 in 2019. Figure 1 shows that rates of opioid overdose deaths among those 55 years and older increased each year from 2000 to 2019, with the largest increase from 2015 to 2016 (1485). From 1999 to 2019, the annual rate of opioid overdose deaths among those 55 years and older increased 10-fold, from 0.90 per 100 000 population in 1999 to a high of 10.70 per 100 000 population in 2019.

**Figure 2.**
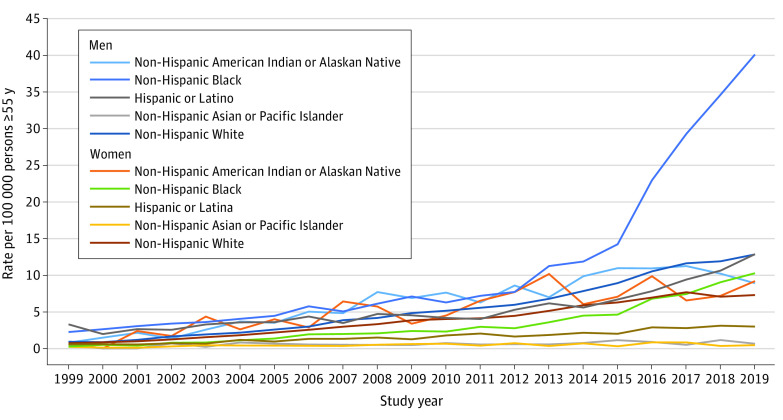
Rates of Opioid Overdose Deaths per 100 000 Persons 55 Years and Older by Sex and by Race and Ethnicity, 1999 to 2019

Next we examined rates of opioid overdose fatalities for adults 55 years and older by the 10 subgroups to determine whether the rates and rate trajectories for the subgroups differed from one another. Figure 2 graphs these results. The Table presents the parameter estimates for the multivariate regression models used to determine the trajectory slopes.

**Table.** Multivariate Regression Parameter Estimates of Linear Trends

Dependent variable	B (SE) [95% CI]^a^	*t* Value^b^
Hispanic or Latina women	1.60 (0.18) [1.22-1.98]	8.84
Hispanic or Latino men	5.25 (0.61) [3.97-6.53]	8.54
Non-Hispanic American Indian or Alaska Native		
Men	6.25 (0.75) [4.67-7.82]	8.28
Women	5.17 (0.63) [3.85-6.48]	8.19
Non-Hispanic Asian or Pacific Islander		
Men	0.58 (0.06) [0.45-0.71]	9.13
Women	0.44 (0.05) [0.35-0.54]	9.70
Non-Hispanic Black		
Men	11.07 (2.41) [6.03-16.10]	4.58
Women	3.22 (0.63) [1.90-4.53]	5.10
Non-Hispanic White		
Men	5.42 (0.84) [3.67-7.17]	6.47
Women	3.84 (0.51) [2.79-4.89]	7.61

^a^ Parameter used was intercept.

^b^ *P* < .001 for all.

We found significant variation in rates of opioid overdose death and rate trajectories by sex and by race and ethnicity. An *F* test of the joint equality of slope parameters across subgroups strongly rejects the null hypothesis of equal trends (*P* < .001). Based on the slope, we identified 3 distinct trajectories. The first trajectory includes those with the lowest slope values (B range, 0.44-1.60). Groups in this trajectory included Hispanic or Latina women and non-Hispanic Asian or Pacific Islander men and women. These demographic groups had the lowest rates of overdose death among this age group, did not experience substantial increases in rates over time, and never exceeded 3 deaths per 100 000 persons.

The second trajectory had a relatively modest slope over time (B range, 3.22-6.25). Groups in this trajectory included Hispanic or Latino men; non-Hispanic American Indian or Alaska Native and non-Hispanic White men; and non-Hispanic American Indian or Alaska Native, non-Hispanic Black, and non-Hispanic White women. The rates for these population segments ranged from 0.23 deaths per 100 000 population in 1999 to 12.84 deaths per 100 000 population in 2019. Although the trajectory slopes of these groups were relatively modest, they suggest a persistent and steady increase in rates of opioid overdose deaths in older adults over time.

The steepest trajectory and outlier was that of non-Hispanic Black men, who had a slope of 11.07, almost twice that of non-Hispanic American Indian or Alaska Native men (6.24). The rates for non-Hispanic Black men ranged from a low of 2.24 deaths per 100 000 population to a high of 40.03 deaths per 100 000 population.

Before 2013, the highest rates in older adults were found among Hispanic or Latino men, non-Hispanic Black men, and non-Hispanic American Indian or Alaska Native men and women. However, in 2013, the rates diverged for non-Hispanic Black men, with steep rate increases for each subsequent year, and this subgroup had the highest rates of any of the 10 subgroups. In 2019, the year with the highest overdose rate for all population segments 55 years and older, the rate of opioid overdose fatalities for non-Hispanic Black men was nearly 4 times higher. Meanwhile, rates for Hispanic or Latino men and American Indian or Alaska Native men and women fluctuated with small increases after 2013.

The rates for Hispanic or Latino men and non-Hispanic White men and women most closely mirrored the overall rate of opioid overdose death for adults 55 years and older from 1999 to 2016. In 2016, men of these groups continued to mirror the overall rate of overdose death for this age group. The rates for non-Hispanic White women essentially remained stable at lower than the overall rate from 2016 from 2019.

## Discussion

Deaths among non-Hispanic Black men appear to account for the disproportionate increase in rates of opioid overdose deaths for all older adults since 2013. This is concerning and may signal future increased rates of opioid overdose deaths for older adults, given that the proportion of older adults who are non-Hispanic Black is growing rapidly compared with non-Hispanic White men.^23^

It is notable that the beginning of the increase in opioid overdose deaths among older non-Hispanic Black men coincides with what the Centers for Disease Control and Prevention^24^ has identified as the third wave in the opioid epidemic characterized by the increased presence of fentanyl, a powerful synthetic opioid in the drug supply. However, it is unclear why other older adult subgroups did not experience concomitant increases in opioid overdose rates. These differences could be, among other factors, related to differences in the characteristics of the drug supply accessed by subgroups and/or whether substances contributing to the overdose were illicit or prescription. Further investigation is needed to elucidate the reasons behind these disparities.

Both racism and ageism may be associated with these disparities. In terms of racism, many potential factors could contribute to higher fatality rates among the non-Hispanic Black population in general and among men in particular. These factors include the consequences of structural racism,^25,26^ such as disparate access to SUD treatment^27^; bias in pain treatment^28^; residential concentration in low-resource communities with limited access to good schools, health care, and healthy foods^29^; medical mistrust^30^; and racially biased drug policies.^31^ In addition, factors associated with ageism and aging complicate our understanding of these trends.

The US Preventive Services Task Force recommends screening adults of all ages for unhealthy alcohol and drug use.^32^ However, screening for substance misuse among older adults is often lacking.^33^ Several factors may contribute to this lack of screening, but ageism in particular reflects widely held beliefs among clinicians and the public that older adults do not develop or experience SUD.^33,34,35^

Another factor may be mistaken attribution of signs of SUD, such as declines in physiological and mental functioning to the process of aging.^33^ Alternatively, aging-related changes in functioning may mask signs of SUD.^33^ In addition, the stigma of SUD, which may be more acute among older adults,^36^ may cause them to hide or fail to disclose substance misuse to clinicians.^33^

Medications for OUD are well established as effective in treating OUD and preventing overdose deaths.^37^ However, less than 10% of older adults admitted for treatment report these medications as part of their OUD treatment plan.^38^ Buprenorphine hydrochloride is the most commonly prescribed agonist for SUD, and it offers distinct advantages for older adults compared with alternatives methadone hydrochloride and naltrexone hydrochloride.^39^ Prescriptions for buprenorphine have grown between 2009 and 2018 for adults aged 55 to 80 years,^40^ but rates of buprenorphine use are still low among older adults seeking OUD treatment.^38^

The news is not all bleak for older adults with SUD. Because older adults use health care more often than younger adults,^23^ and owing to recent changes in health care coverage (including the Affordable Care Act and Medicaid expansion) that have made health care more accessible to all, opportunities for SUD screening in health care settings are increasing. To take advantage of this opportunity, expanded training is needed for physicians, advanced nurse practitioners, nurses, home health care workers, and other clinicians as well as other elder support service professionals who supply meal home delivery and transportation services. In addition, training in recognition of the signs of SUD in older adults, referral to SUD screening, and implementation of SUD screening as recommended by the US Prevention Task Force are needed.^32^

There is evidence, at least for alcohol use disorder, that older adults are amenable to alcohol use treatment, particularly if age-specific services are available.^33^ However, it is not clear whether this acceptance of treatment is transferrable to older adults with nonalcohol SUDs.

Once in treatment, older persons with SUD are more often successful in their recovery, compared with middle-aged and younger adults.^41^ One possible reason for this increased success compared with younger persons may be related to cognitive functions and social emotional intelligence traits associated with aging.^41,42^ Traits such as wisdom, complex decision-making skills, emotional regulation, and self-reflection may increase the likelihood of successful completion of SUD treatment. However, evidence suggests that cognitive impairment is common among older adults with SUD,^43^ and as a result, many older adults may not experience the cognitive or emotional advantages of being an older adult in SUD treatment.

The uniqueness of older adults with SUD suggests that for SUD treatment, as with most forms of health care, a one-size-fits-all model is not likely to provide optimum success. Some evidence suggests that older adults are more successful with higher-dosage treatment models, but more information is needed to match older adult needs with treatment modalities.^43,44^ Treatment for SUD must take into account issues unique to older adults, and more specifically among older non-Hispanic Black men with SUD. For example, it has been reported that older adults are less comfortable attending group therapy than younger adults.^45^ It is speculated that this difference may be due in part to generational prohibitions against discussing trauma and internalized stigma regarding substance misuse.^23^ Specific to non-Hispanic Black men, issues of mistrust and experiences of racism will need to inform treatment models. This may require use of credible messengers (eg, those with similar lived experience) to achieve outreach and treatment goals. We use the concept of a credible messenger adapted from the Cure Violence prevention program model.^46^ Credible messengers can relate to the target population. They are considered credible because they are a part of the community being served, can relate to high-risk individuals, are respected by high-risk individuals, and have the ability to engage, connect, and empathize with them. In most cases, credible messengers have relevant lived experience and are therefore seen as having “been there and done that” with regard to risk behaviors, which allows them to reach the target population in ways that others cannot. Both outreach workers and violence interrupters should be credible messengers, allowing them to reach the target population in ways that others cannot. Other considerations for tailoring SUD treatment for older adults include the medical complexity of older adults who disproportionately experience physical health comorbidities and polypharmacy, which may interact with their SUD recovery, accessibility for those with limited mobility, transportation needs, and accommodations for hearing and vision impairments^36,39^

Our findings on sex and racial and ethnic disparities in opioid overdose deaths among older adults offer a starting point for further investigation. Further research is needed to address the reasons behind the increase in opioid overdose deaths in older adults and the factors associated with racial disparities. For example, more information is needed regarding older non-Hispanic Black men who die due to opioid overdose, including substance misuse history, presence of a diagnosed SUD, past treatment episodes,^47^ presence of medical conditions, social isolation, and mental health disorders. This information can help to inform intervention, treatment, and prevention approaches for older adults with SUD.

### Limitations

This study has some limitations. As a retrospective study, it uses data from death certificate and medical examiner reports, which may include inaccuracies. In addition, these individuals may have multiple causes of death beyond opioids not considered in this study. Last, we lack detailed clinical, circumstantial, and fatal incident variables that could better characterize the context of these deaths.

## Conclusions

This longitudinal cross-sectional study found that opioid overdose fatality rates for US adults 55 years and older increased substantially since 1999. That burden was experienced differentially by sex and race and ethnicity. In recent years, risk was most concentrated among non-Hispanic Black men. Beginning in 2013, the rate of opioid overdose deaths for non-Hispanic Black men was substantially higher than the overall rate for persons 55 years and older and the rates for other subgroups that were examined in this study.
